# *Phlebotomus (Paraphlebotomus) chabaudi* and *Phlebotomus riouxi*: closely related species or synonyms?

**DOI:** 10.1051/parasite/2017050

**Published:** 2017-12-01

**Authors:** Véronique Lehrter, Anne-Laure Bañuls, Nicole Léger, Jean-Antoine Rioux, Jérôme Depaquit

**Affiliations:** 1 EA 4688 - USC ANSES VECPAR, SFR Cap Santé, UFR de Pharmacie, Université de Reims Champagne-Ardenne, 51 rue Cognacq-Jay, 51096 Reims France; 2 UMR MIVEGEC, IRD - CNRS - Université de Montpellier, 911 avenue Agropolis, 34394 Montpellier France; 3 Faculté de Médecine, Université de Montpellier, 2 rue de l'École de Médecine, 34000 Montpellier France

**Keywords:** *Phlebotomus chabaudi*, *Phlebotomus riouxi*, mitochondrial, nuclear and ribosomal markers, phylogenetic, North Africa

## Abstract

*Phlebotomus riouxi* Depaquit, Killick-Kendrick & Léger 1998 was described as a species closely related to *Phlebotomus chabaudi* Croset, Abonnenc & Rioux 1970, differing mainly by the size and number of setae of the coxite basal lobe. Molecular studies carried out on several populations from Algeria and Tunisia and based on mitochondrial genes cytochrome b (Cytb) and cytochrome oxidase I (COI) supported the typological validity of these two species. Recently, specimens from a single population in southern Tunisia were morphologically identified as *Ph. riouxi*, *Ph. chabaudi* and intermediates, but were clustered in the same clade according to their Cytb and nuclear gene elongation factor-1 α (EF-1α) sequences. These species were thus synonymized. To further explore this synonymy, we carried out a molecular study on specimens from Algeria and Tunisia using the same molecular markers and a part of 28S rDNA. We did not find any morphologically intermediate specimens in our sampling. We highlighted differences between the genetic divergence rates within and between the two species for the three markers and we identified new haplotypes. The sequence analysis did not reveal any signature of introgression in allopatric nor in sympatric populations such as in the Ghomrassen population. Phylogenetic analyses based on our specimens revealed that the two main clades are *Ph. chabaudi* and *Ph. riouxi*, in agreement with the morphological identification. These results support the validity of *Ph. riouxi* and *Ph. chabaudi* as typological species.

## Abstract (fr)

*Phlebotomus riouxi* Depaquit, Killick-Kendrick & Léger 1998 a été décrit comme une espèce proche de *Phlebotomus chabaudi* Croset, Abonnenc & Rioux 1970, se distinguant principalement par la taille et le nombre de soies sur le lobe basal du coxite. Des études moléculaires, menées sur plusieurs populations d'Algérie et de Tunisie, et basées sur les gènes mitochondriaux cytochrome b (Cytb) et cytochrome oxydase I (COI), ont soutenu la validité typologique de ces deux espèces. Récemment, des spécimens d'une seule population du sud de la Tunisie ont été morphologiquement identifiés comme des *Ph. riouxi*, *Ph. chabaudi* et intermédiaires, mais se sont retrouvés groupés dans le même clade selon leurs séquences de Cytb et de facteur d'élongation 1 alpha (EF-1α). Ces espèces ont donc été mises en synonymie. Afin d'explorer davantage cette synonymie, nous avons mené une étude moléculaire sur des spécimens d'Algérie et de Tunisie en utilisant les mêmes marqueurs moléculaires ainsi qu'une partie du 28S de l'ADN ribosomique. Aucun spécimen ne présentait de morphologie intermédiaire dans notre échantillonnage. Des différences entre les taux de variabilité génétique intra et interspécifiques des trois marqueurs ont été mises en évidence, ainsi que de nouveaux haplotypes. L'analyse des séquences n'a révélé aucune signature d'introgression que ce soit dans les populations allopatriques ou sympatriques telle que la population de Ghomrassen. Les analyses phylogénétiques basées sur nos échantillons ont révélé que les deux principaux clades correspondent à *Ph. chabaudi* et *Ph. riouxi*, résultat en accord avec l'identification morphologique. Ces résultats soutiennent la validité de *Ph. riouxi* et *Ph. chabaudi* comme espèces typologiques.

## Introduction

Within the *Phlebotomus* genus (Diptera, Psychodidae), the subgenus *Paraphlebotomus* Theodor 1948 includes some proven and suspected vectors of leishmaniases, e.g. *Phlebotomus sergenti*, the main vector of *Leishmania tropica* [2,20]. Our study focuses on two species of *Paraphlebotomus* from North Africa: *Phlebotomus chabaudi* Croset, Abonnenc & Rioux 1970 and *Phlebotomus riouxi* Depaquit, Killick-Kendrick & Léger, 1998 [9,10,35]. The presence of *Ph. chabaudi* has also been reported in southern Spain [34].

Although their vectorial role has never been demonstrated, these two species are recorded in several leishmaniasis foci [3,28,40] and are related to *Ph. sergenti*. In fact, *Ph. chabaudi* and *Leishmania killicki* have been described for the first time in the same locality (Tataouine) in Tunisia [36], and *L. killicki* was also found in Algeria [24,26], especially in Ghardaïa, where some *Ph. riouxi* were reported, even though *Ph. sergenti* was the main proven vector [4].

In previous studies, *Ph. chabaudi* and *Ph. riouxi* males collected in Algeria and Tunisia were clearly identified morphologically. Molecular processing used two mitochondrial genes: a partial sequence of cytochrome b (Cytb-CB3) [6], as proposed by Esseghir *et al.* [17], and cytochrome oxidase 1 (COI) [5]. In both studies, phylogenetic analyses emphasized the validity of the two species, supporting their typological status, meaning that the deposited type-specimens are fully justified.

Recently, several specimens from Southern Tunisia showed ambiguous morphological characters [40,41]. According to these authors, several morphological criteria described as specific characters were found together in some specimens that they described as intermediate specimens. They used the same mitochondrial marker as that of Bounamous *et al.* [6], called Cytb-CB3, in order to compare their sequences with those available in GenBank. They also sequenced a longer fragment of Cytb (called Cytb-CB) and the nuclear elongation factor-1alpha gene (EF-1α) [30,41]. Their molecular results did not match with the morphological identification, not only for the intermediate specimens, but also for the differentiation between *Ph. chabaudi* and *Ph. riouxi*: all specimens were clustered in the same clade. According to these results, based on specimens from the single locality of Ghomrassen, they proposed to consider *Ph. riouxi* as a junior synonym of *Ph. chabaudi*.

In order to better understand the situation, we decided to broaden the approach by performing a comparative and combined sequence analysis of three loci on larger samples from different geographical populations we previously investigated. We included the two markers used by Tabbabi's team [40,41], Cytb-CB and nuclear EF-1α, and the D1-D2 domain of ribosomal 28S DNA which is known as a good marker for studying the interspecific genetic divergence between species [19,22,38]. This domain has specifically been used to perform analysis at the taxonomic level in Phlebotominae [11,13,31].

## Material and methods

### Sample collection

**Figure 1:**
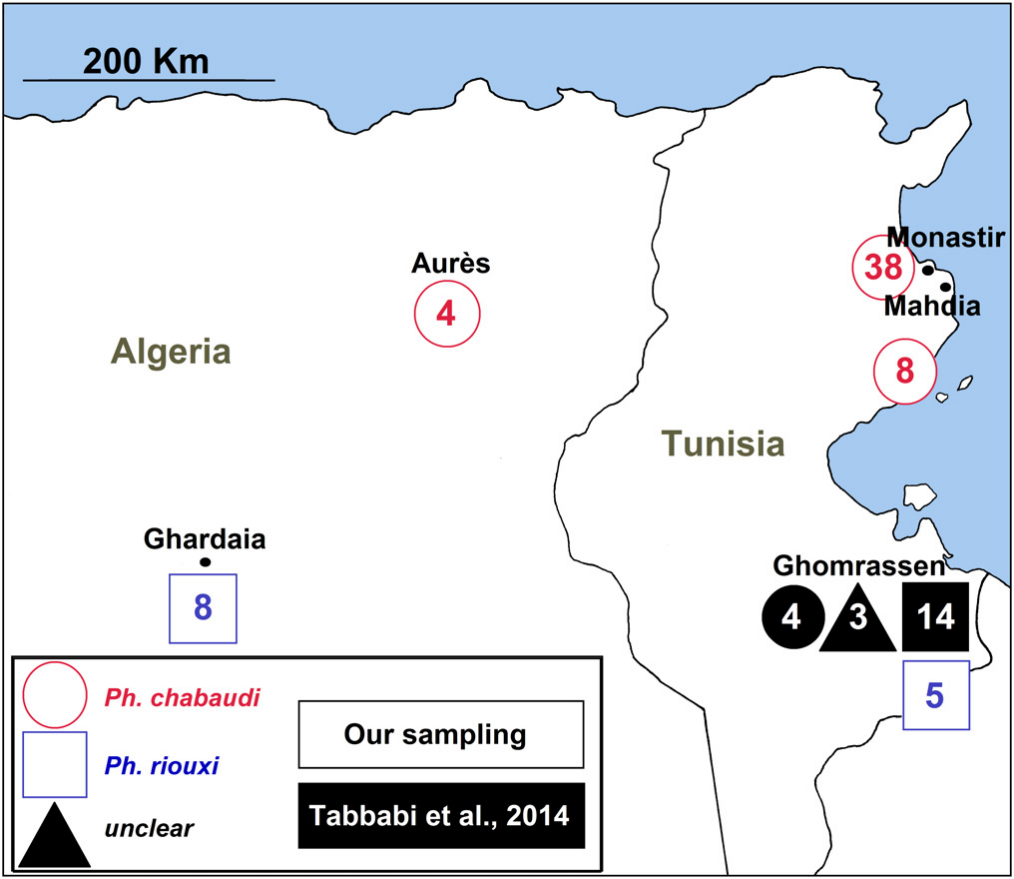
Sampling locations. Numbers indicate the number of specimens studied with round and square symbols corresponding to *Ph. chabaudi* and *Ph. riouxi*, respectively. Samples with a black fill come from Tabbabi's sampling while those with a white fill come from our sampling. The three uncertain specimens of Tabbabi are indicated by a rounded square.

Samples analyzed in the present study were those used by Tabbabi *et al.*, Bounamous *et al.* and Boudabous *et al.* [5,6,40,41]. For the Tabbabi samples included in this analysis, we only had access to published data. All the other specimens came from our laboratory, including samples used by Bounamous *et al.* and Boudabous *et al.* [5,6], for which we kept the same sample codes marked in bold in Tables 1 and 3. Our specimens were collected by CDC miniature light traps and sticky paper traps from two regions of Algeria (Ghardaïa and Aurès) and from three regions of Tunisia (Mahdia, Monastir and Ghomrassen) (see Table 1 and Figure 1).

**Table 1:**
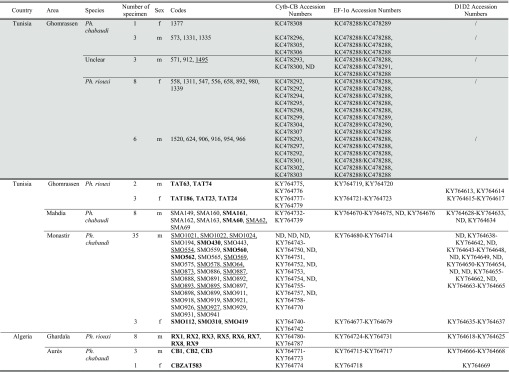
Analyzed samples. On a grey background, the samples processed by Tabbabi's team. Samples in bold were used by Bounamous *et al.* and Boudabous *et al.* [5,6]. Samples not sequenced with all markers are underlined and their accession number replaced by ND.

**Table 3:**
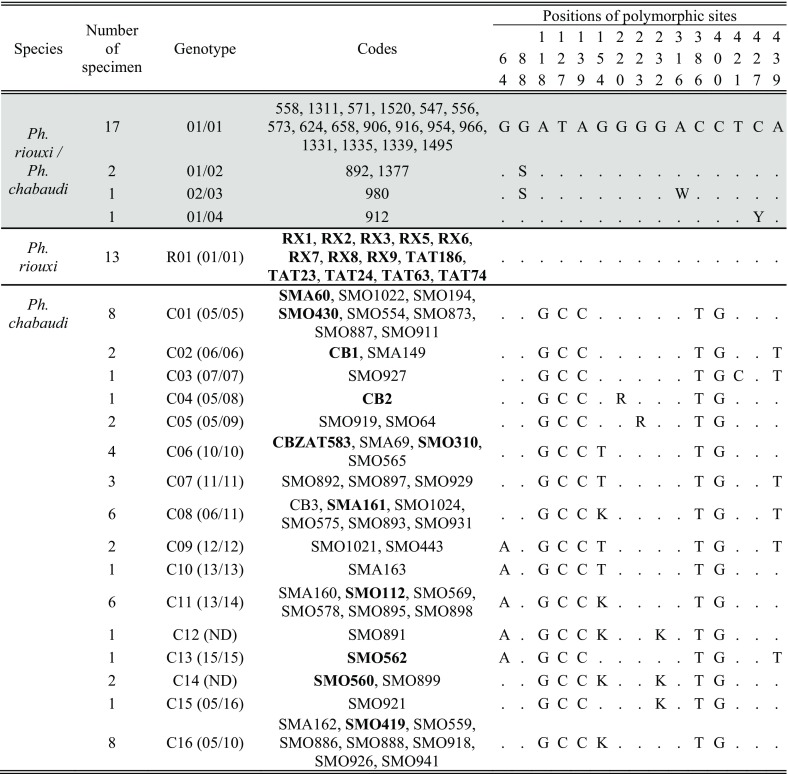
Base variability in EF-1α genotypes. On a grey background: four alleles found by Tabbabi *et al.* (2014) (01 to 04 = KC478288 to KC478291). On a white background: 16 new genotypes found in our sample (n = 62), composed of 12 new haplotypes (05 to 16); ND = Non Determinate. Samples in bold were used by Bounamous *et al.* and Boudabous *et al.* [5,6].

Specimens were stored in 95% ethanol at −20 °C, until dissection. After thawing, each specimen was dissected individually in 95% ethanol with sterile needles. The head and the genitalia were cleared in boiled Marc André solution and mounted in chloral gum between the slide and cover slide for microscopic observation. The rest of the body was dried and preserved at −20 °C in a sterile microtube until DNA extraction.

Taking into consideration the difficulty in identifying females, we restricted the number of females in our sampling. The 9 females and 12 males of Tabbabi's study [41] are represented in Figure 1 as black symbols. Unfortunately, we did not have access to these specimens. However, all the specimens studied by Bounamous *et al.* and Boudabous *et al.* [5,6], as well as the new samples, were morphologically examined or re-examined in the present study, according to the criteria previously described [6,9,10].

**Figure 2:**
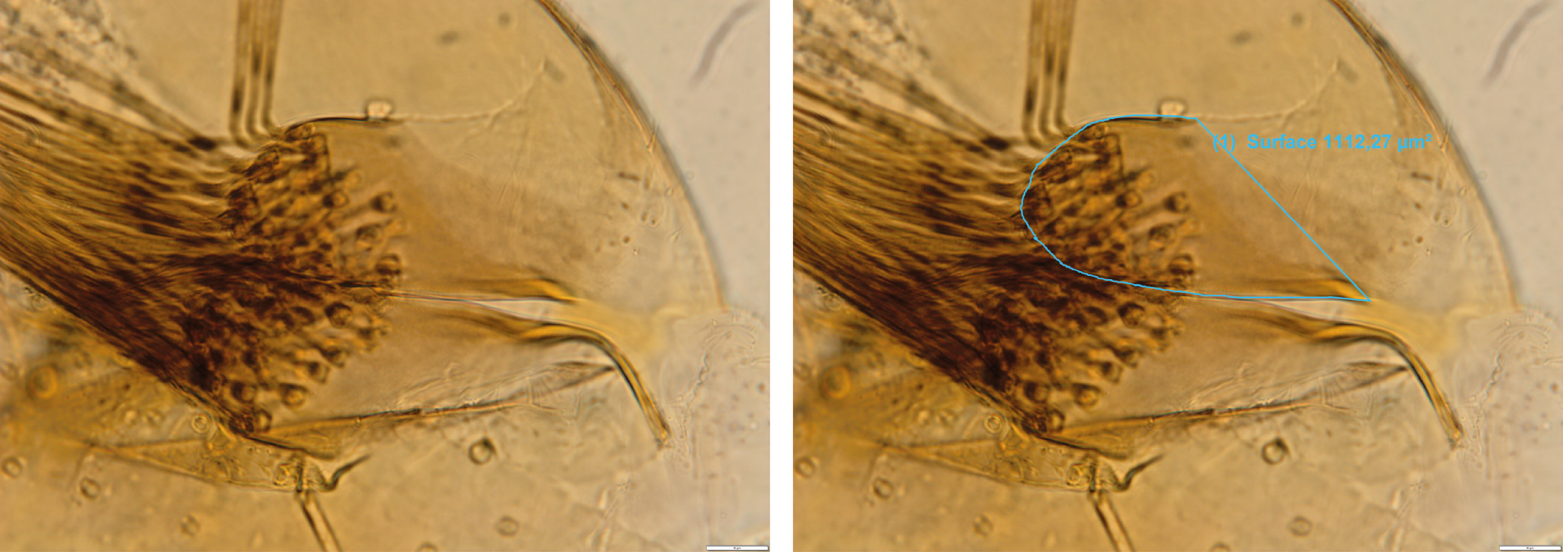
Illustration of the procedure of morphometrical measures of area and perimeter of the basal lobe of the coxite. Left, unmarked; right, marked with perimeter and area.

The morphological analyses were focused on the basal lobe of the coxite, known to be the differential character between the two species under study [6,10]. For each processed specimen, the number of coxite lobe setae was counted and morphometrics analysis, using Stream Motion 1.9.1 software (Olympus, Japan), was also applied. In 1998, it was suggested that the number of setae, and the width and length of the coxite lobe were informative to differentiate the species [10]. The width measure did not cause any difficulty: a transversal line was perpendicularly traced in the larger part of the lobe. Considering the difficulties in measuring the length of the basal lobe of the coxite, we substituted it by its perimeter and area, as indicated in Figure 2.

### DNA extraction, PCR and sequence analysis

Some DNA extracts (sample codes in bold in Tables 1 and 3) from previous studies [5,6] were simply thawed for direct PCR amplification. For the other specimens, we used the same procedure as for the older extracts: DNA extractions were individually carried out using a QIAamp DNA Mini Kit (Qiagen), according to the manufacturer's protocol. Samples were crushed in ATL buffer with a piston pellet and DNA extracts were eluted in 180 μL to 200 μL of AE buffer and stored at −20 °C. Cytb-CB was amplified using CB1-SE: 5′-TATGTACTACCCTGAGGACAAATATC-3′ and CB-R06: 5′-TATCTAATGGTTTCAAAACAATTGC-3′ primers, as previously described [41]. For EF-1α amplification, primers EF-F05: 5′-CCTGGACATCGTGATTTCAT-3′ and EF-R08: 5′-CCACCAATCTTGTAGACATCCTG-3′ were used [41]. The ribosomal domain D1-D2 was amplified using C1′: 5′-ACCCGCTGAATTTAAGCAT-3′and D2: 5′-TCCGTGTTTCAAGACGGG-3′ primers [32]. The PCR conditions for Cytb-CB and EF-1α were exactly the same as those used by Tabbabi *et al.* [41]. All PCRs were performed in a 50 μL volume using 5 μL of extracted DNA solution of each specimen individually, and 12.5 pmol of the primer sets in a thermocycler. The PCR mix contained (final concentrations) 10 mM Tris HCl (pH 8.3), 1.5 mM MgCl_2_, 50 mM KCl, 0.01% Triton X-100, 200 mM dNTP each base, and 1.25 units of Taq polymerase (Eppendorf, Germany). For each PCR run, a negative control using 5 μL of ultrapure sterile water and a positive control using a DNA extract with a known sequence, were included. PCR programs were carried out with an initial denaturation step at 94 °C for 3 min and finished by an extension step at 68 °C for 10 min. Between these two steps, cycling program parameters depended on the markers: for Cytb-CB, 40 cycles of denaturation at 94 °C for 45 sec, annealing at 50 °C for 45 sec, and extension at 68 °C for 1 min; for D1-D2, 30 cycles of denaturation at 94 °C for 1 min, annealing at 58 °C for 1 min, and extension at 68 °C for 1 min; for EF-1α, 5 cycles of denaturation at 94 °C for 30 sec, annealing at 44 °C for 30 sec, and extension at 68 °C for 1 min, followed by 30 similar cycles with an annealing temperature at 48 °C [29]. All PCR products were first verified using the molecular weight marker 100 bp DNA Ladder (Promega) in GelGreen (Biotium) stained 1.5% agarose gel electrophoresis. The PCR products were then sequenced in both directions on a Beckman Coulter Genomics sequencer, with the same primers used for PCR except that CB1-SE was replaced by CB1: 5′-TATGTACTACCATGAGGACAAATATC-3′, as mentioned by Tabbabi *et al.* [41]. Sequences were then aligned with the alignment editor implemented in BioEdit 7.0.8.0 [23] and checked by eye. Sequence alignments were performed respecting the following criteria: (1) to minimize the number of inferred mutations (number of steps); (2) to prefer substitution to insertion-deletion, and (3) to prefer transitions to transversions. Genetic divergences between the sequences were measured using the Tamura-Nei model and the presence of open reading frames (ORFs) was checked using MEGA6 [42]. Tabbabi's sequences were used as the reference to perform alignment. The primer sequences were removed. Phylogenetic inferences derived from maximum likelihood (ML) for each gene separately were performed using the PhyML 3.0 software program [21]. For these analyses, the best fitting nucleotide substitution model was determined through the automatic model selection tool available on the PhyML server. Then, we used RAxML software [39] for a partitioned ML analysis with a GTR (general time reversible) model for the combined analysis of the three genes. Each gene was considered as a different partition and a specific separate substitution model was assigned. For each analysis, bootstrapping was used to test the branch strength of the phylogenetic trees. For all phylogenies, we used one sequence of *Ph. sergenti* as the outgroup. The trees were then visualized using TreeDyn, version 198.3 [7]. Whenever possible, sequences of *Ph. chabaudi* published by Tabbabi *et al.* [41] were added to the analyses.

## Results

### Morphological identification

All of our specimens were correctly identified as *Ph. chabaudi* and *Ph. riouxi*, according to morphological criteria of initial descriptions for males [9,10] and to the pharyngeal armature for females [6]. All females were morphologically identified based on the presence or absence of anterolateral teeth in the pharynx. All measures related to the coxite lobe are listed in Table 2. Considering these data, we found significant interspecific differences between all measurements of the basal lobe of the coxite, without significant geographical variability (data not shown). We did not find any morphological intermediate specimen. All *Ph. riouxi* were found in the south of Tunisia and in the south of Algeria.

**Table 2:** Morphological data of male samples: for each species, mean and standard deviation (sd) are calculated and minimal (min) and maximal (max) values are indicated.

Species		Coxite lobe area (μm^2^)	Coxite lobe perimeter (μm)	Coxite lobe width (μm)	Number of setae per coxite lobe
*Ph. riouxi*	**mean**	**1154**	**160**	**27**	**35**
	sd	118	7	3	5
	min	926	148	23	28
	max	1331	167	32	43
*Ph. chabaudi*	**mean**	**415**	**95**	**14**	**11**
	sd	94	11	2	2
	min	209	65	10	7
	max	609	116	18	17

### Sequence analyses

A total of 63 specimens were analyzed and compared to the sequences of the 21 specimens from Tabbabi *et al.* [41] (Table 1).

#### Cytochrome b analyses

**Figure 3:**
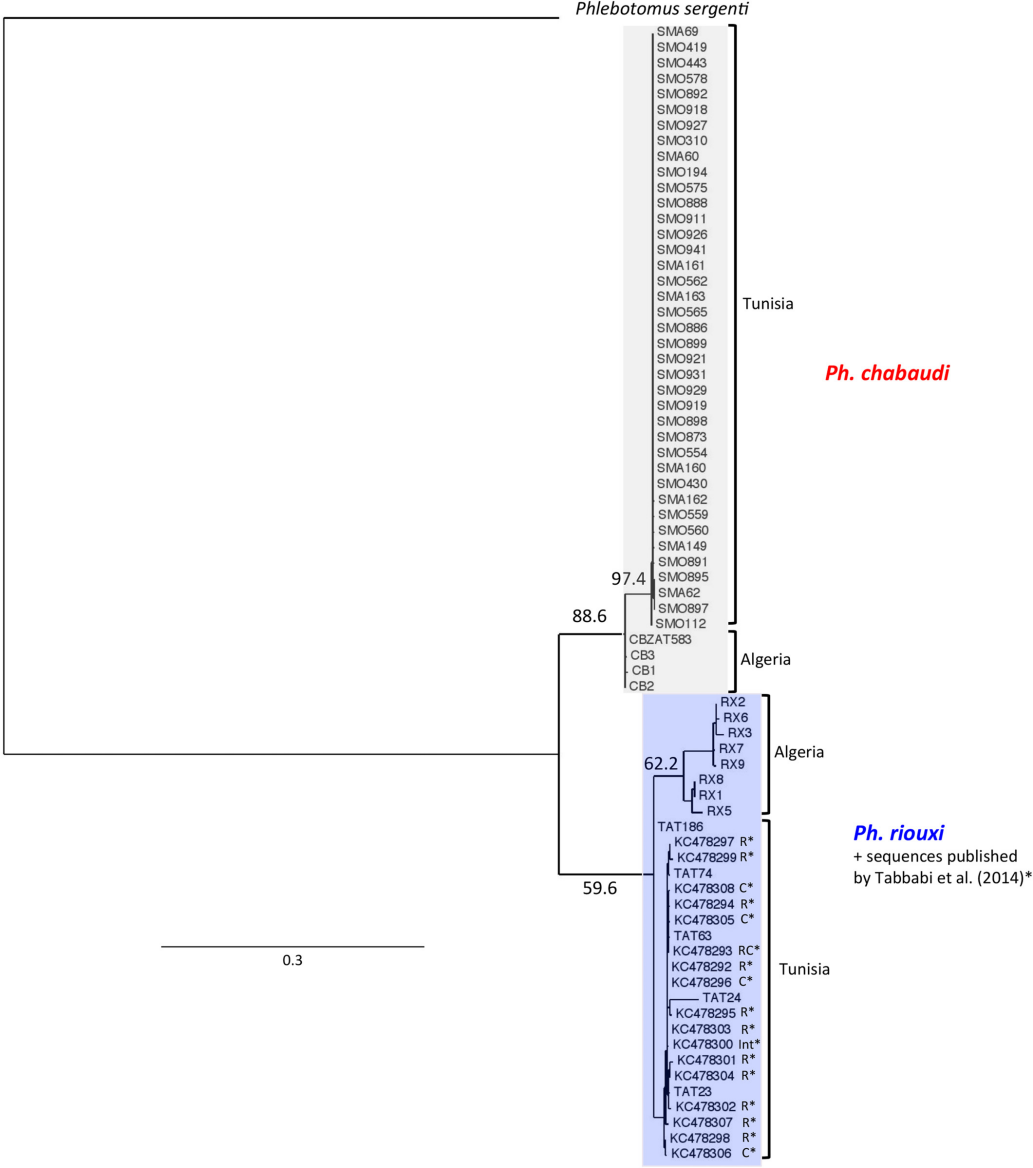
Phylogenetic tree inferred from cytochrome B data of *Phlebotomus chabaudi* and *Ph. riouxi* specimens. We added to the analysis the sequences of *Ph. chabaudi* published by Tabbabi *et al.* (2014). The phylogram results from bootstrapped data sets obtained using the PhyML 3.0 program [21] using GTR (general time reversible) + Γ distribution (gamma distribution of rates with four rate categories). The tree was visualized using the TreeDyn program, version 198.3 [7]. The percentages above the branches are the frequencies with which a given branch appeared in 500 bootstrap replications. Only bootstrap values higher than 50% on the early branches are shown. A sequence of *Ph. sergenti* (AF161216) was used as the outgroup. The sequences marked by * were published by Tabbabi *et al.* (2014); R = sequences found in specimens morphologically characterized as *Ph. riouxi*. C = sequences found in specimens morphologically characterized as *Ph. chabaudi*. RC = sequences found in specimens morphologically characterized as *Ph. chabaudi* or Ph. riouxi. Int = sequences found in specimens morphologically characterized as intermediate between *Ph. riouxi* and *Ph. chabaudi*.

For the 56 specimens successfully amplified, the length of the cytochrome b fragment Cytb-CB used for analysis was 628 bp. The phylogenetic tree obtained by adding 17 haplotypes of Tabbabi's data [41] highlighted two distinct clades (Figure 3). The first one was only composed of *Ph. chabaudi* as supported by a bootstrap value of 88.6%, with a geographical subdivision between Algeria (bootstrap value < 50%) and Tunisia (bootstrap value = 97.4%). The second clade was composed of a mix of our own *Ph. riouxi* and the 17 haplotypes of Tabbabi from the Ghomrassen area, identified as *Ph. chabaudi*, *Ph. riouxi* and intermediates. For this clade, the bootstrap value was lower (59.6%). Within this clade, the specimens were also subdivided into two clusters according to the geographic area, i.e. southern Tunisia versus southern Algeria but with low bootstrap values < 50% and = 62.2 %, respectively.

We obtained congruent results between morphology and molecular observations in our samples, with an interspecific genetic divergence of 12.2% (SD = 1.38%) for Cytb-CB between *Ph. chabaudi* and *Ph. riouxi*. Eleven haplotypes in our 43 *Ph. chabaudi* samples had an intraspecific divergence of 0.60% (SD = 0.13%), and interestingly, a higher intraspecific divergence in the 13 *Ph. riouxi* with 12 haplotypes: 4.38% (SD = 0.59%).

#### EF-1α analyses

**Figure 4:**
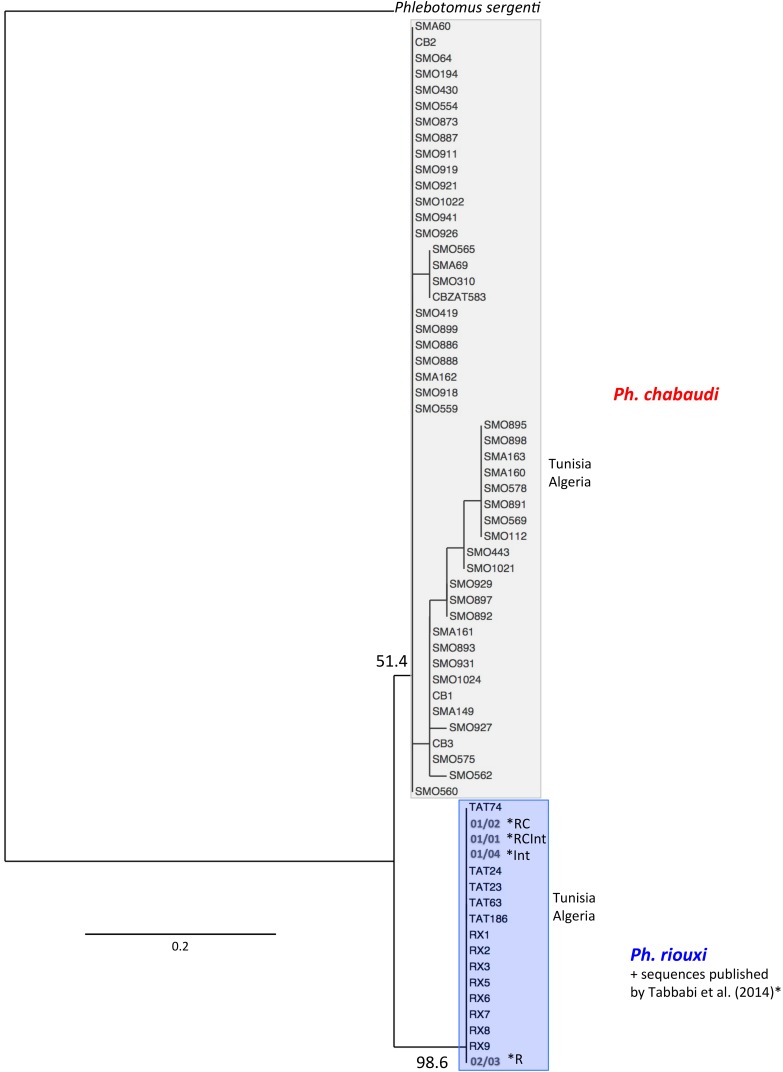
Phylogenetic tree inferred from *Phlebotomus chabaudi* and *Ph. riouxi* specimens using the data of elongation factor 1-α gene. Sequences of *Ph. chabaudi* published by Tabbabi *et al.* (2014) were added to the analyses. The phylogram results from bootstrapped data sets obtained using the PhyML 3.0 program [21] using the HKY85 [25] + I (proportion of invariant sites) model. The tree was visualized using the TreeDyn program, version 198.3 [7]. Percentages shown above the branches are the frequencies at which a given branch appeared in 500 bootstrap replications. Only bootstrap values higher than 50% on the early branches are shown. A sequence of *Ph. sergenti* (EF416841) was used as the outgroup. The sequences marked by * were published by Tabbabi *et al.* (2014); R = sequences found in specimens morphologically characterized as *Ph. riouxi*. RC = sequences found in specimens morphologically characterized as *Ph. chabaudi* or *Ph. riouxi*. Int = sequences found in specimens morphologically characterized as intermediate between *Ph. riouxi* and *Ph. chabaudi*. RCint = sequences found in specimens morphologically characterized as *Ph. riouxi*, *Ph. chabaudi* and intermediate specimens between the two species.

EF-1α amplification gave a fragment of 454 bp in length. All variable sites of 83 sequences alignment are shown in Table 3. One genotype R01 (01/01) was found in all 13 specimens we identified as *Ph. riouxi*. They corresponded to genotype 01/01 of Tabbabi's samples (=genotype R01 according to our label). Interestingly, *Ph. chabaudi* showed very different genotypes (C01 to C16) compared to R01, with five different bases between the two clades, as seen in the phylogenetic tree based on genotypes (Figure 4). These data led to a high bootstrap value for the *Ph. riouxi* clade (98.6%) and a lower one (51.4%) for *Ph. chabaudi*.

Analyses considering genotypes were performed as we obtained several heterozygous positions (double peaks), represented by ambiguous bases in the sequences, for three specimens (Table 3). Indeed, several ambiguous positions were detected in some specimens in the same sequence, precluding the haplotype determination. This is the case for genotypes C12 and C14, corresponding to three *Ph. chabaudi*. All double peaks were located in the third position of the codon without changing the amino acid translation. Overall, on the 46 samples for which the haplotypes could be determined, the frequency of the major haplotype was 30% (haplotype 05), followed by haplotype 10 and haplotype 06 with 17% and 11%, respectively. Concerning our sampling, interspecific divergence of EF-1α between *Ph. riouxi* and *Ph. chabaudi* was 1.15% (SD = 0.53%), and intraspecific divergences were 0 and 0.009%, respectively, corresponding to normal values for this marker [43].

Conversely to Tabbabi's studies [40,41], the sequences obtained in our sampling were congruent with the morphological identification.

#### D1-D2 analyses and concatenate analyses

**Figure 5:**
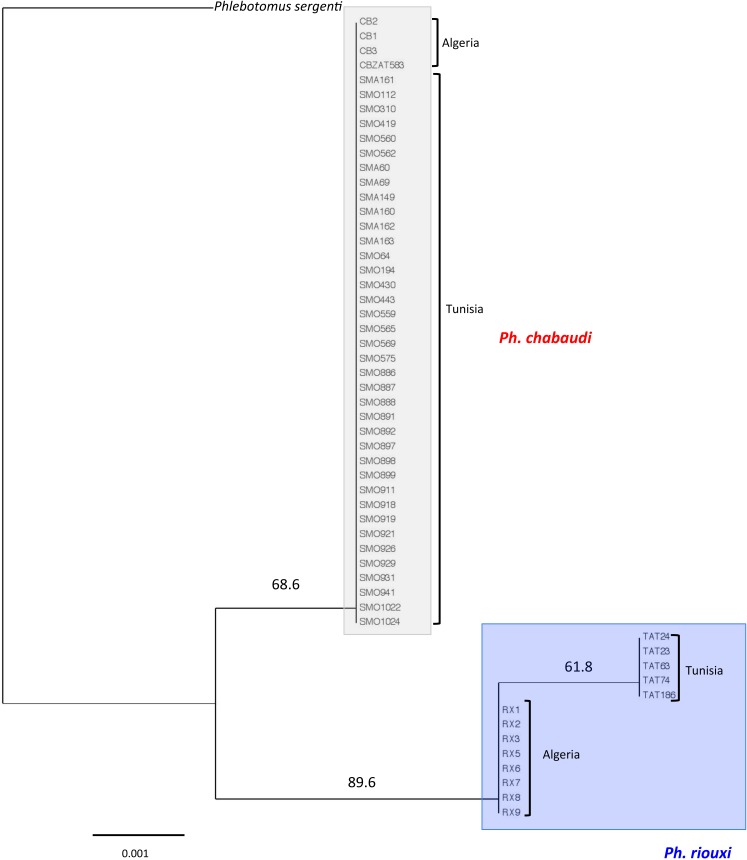
Phylogenetic tree inferred from *Phlebotomus chabaudi* and *Ph. riouxi* specimens using the data of D1-D2 domain of 28S rDNA. Sequences of *Ph. chabaudi* published by Tabbabi *et al.* (2014) were added to the analyses. The phylogram results from bootstrapped data sets obtained using the PhyML 3.0 program [21] using the HKY85 model [25]. The tree was visualized using the TreeDyn program, version 198.3 [7]. The percentages above the branches are the frequencies with which a given branch appeared in 500 bootstrap replications. Only bootstrap values higher than 50% on the early branches are shown. A sequence of *Ph. sergenti* (KY764627) was used as the outgroup.

D1-D2 amplification of our 55 samples gave fragments from 712 to 714 bp in length. For this marker, the interspecific genetic divergence between *Ph. chabaudi* and *Ph. riouxi* was 0.50% (SD = 0.26%) and the intraspecific divergence was 0 and 0.1%, respectively. This value (0.50%) was close to the genetic divergence observed between the two well-separated species *Ph. chabaudi* and *Ph. sergenti* (0.74%, SD = 0.31%). The lack of or the very low intraspecific divergence can be explained by the low mutation rate of this conserved marker [19,22,38]. Phylogenetic analysis based on the sequences of the D1-D2 domain of 28S rDNA allowed us to differentiate the two species by their clustering into two main clades (Figure 5). In spite of a low genetic divergence, the bootstrap value was strong for the *Ph. riouxi* clade (89.6%) and 68.6% for *Ph. chabaudi.*

**Figure 6:**
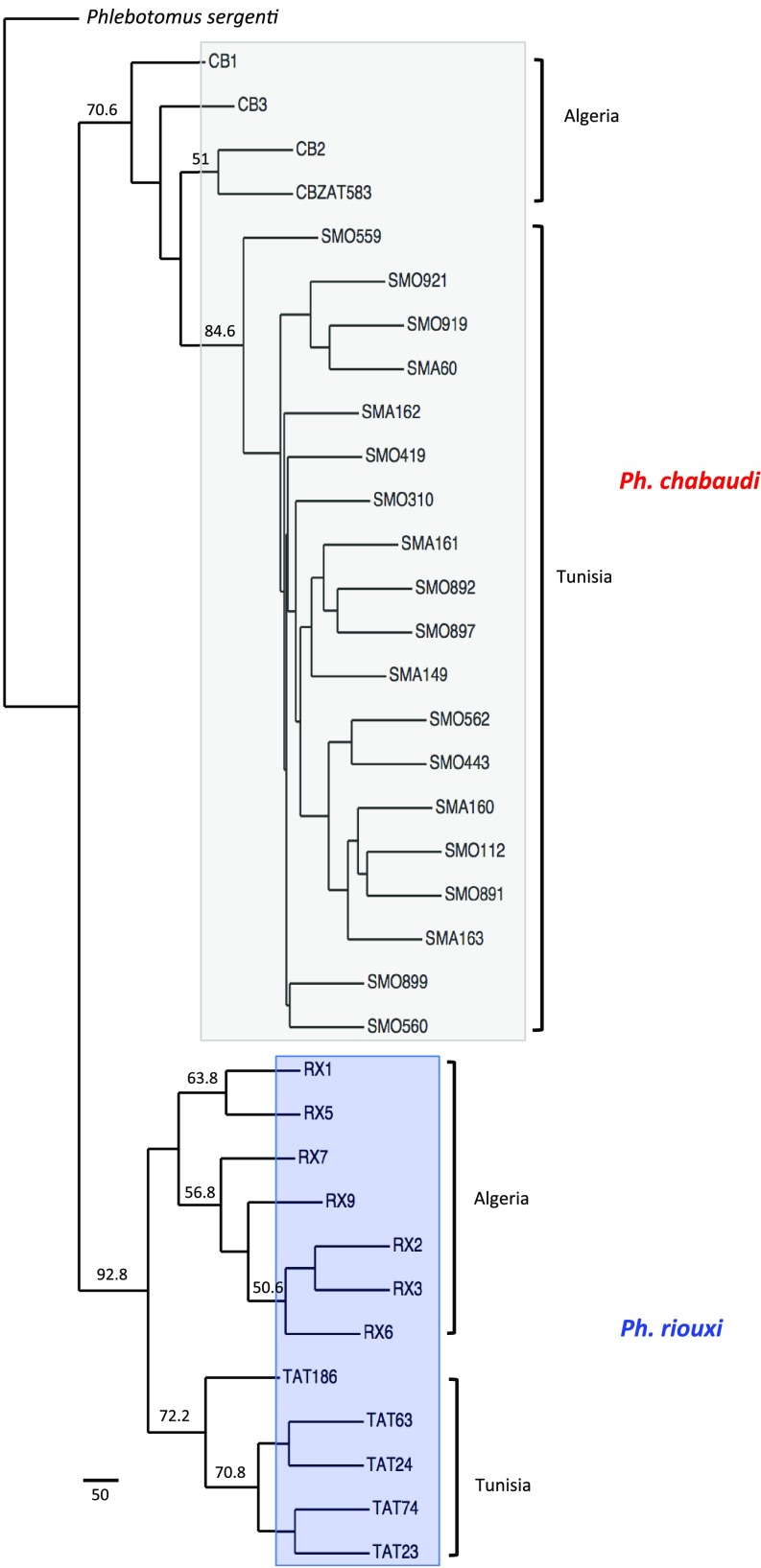
Phylogenetic tree inferred by concatenation of the three loci under study. The phylogram was obtained by a partitioned ML analysis with a GTR (general time reversible) + Γ (gamma distribution of rates with four rate categories) + I (proportion of invariant sites) model using RAxML software [39]. The tree was visualized using the TreeDyn program, version 198.3 [7]. The percentages above the branches are the frequencies with which a given branch appeared in 500 bootstrap replications. Only bootstrap values higher than 50% on branches are shown. Concatenated sequences of *Ph. sergenti* were used as the outgroup.

Concatenated analyses of the three loci using a partitioned ML model also showed clear clustering in two clades corresponding to the morphological identifications of *Ph. chabaudi* and *Ph. riouxi* (Figure 6). The bootstrap values were 70.6% for the *Ph. chabaudi* clade and 92.8% for the *Ph. riouxi* clade. As indicated by the comparison of the trees, we did not find any signs of introgression.

## Discussion

**Figure 7:**
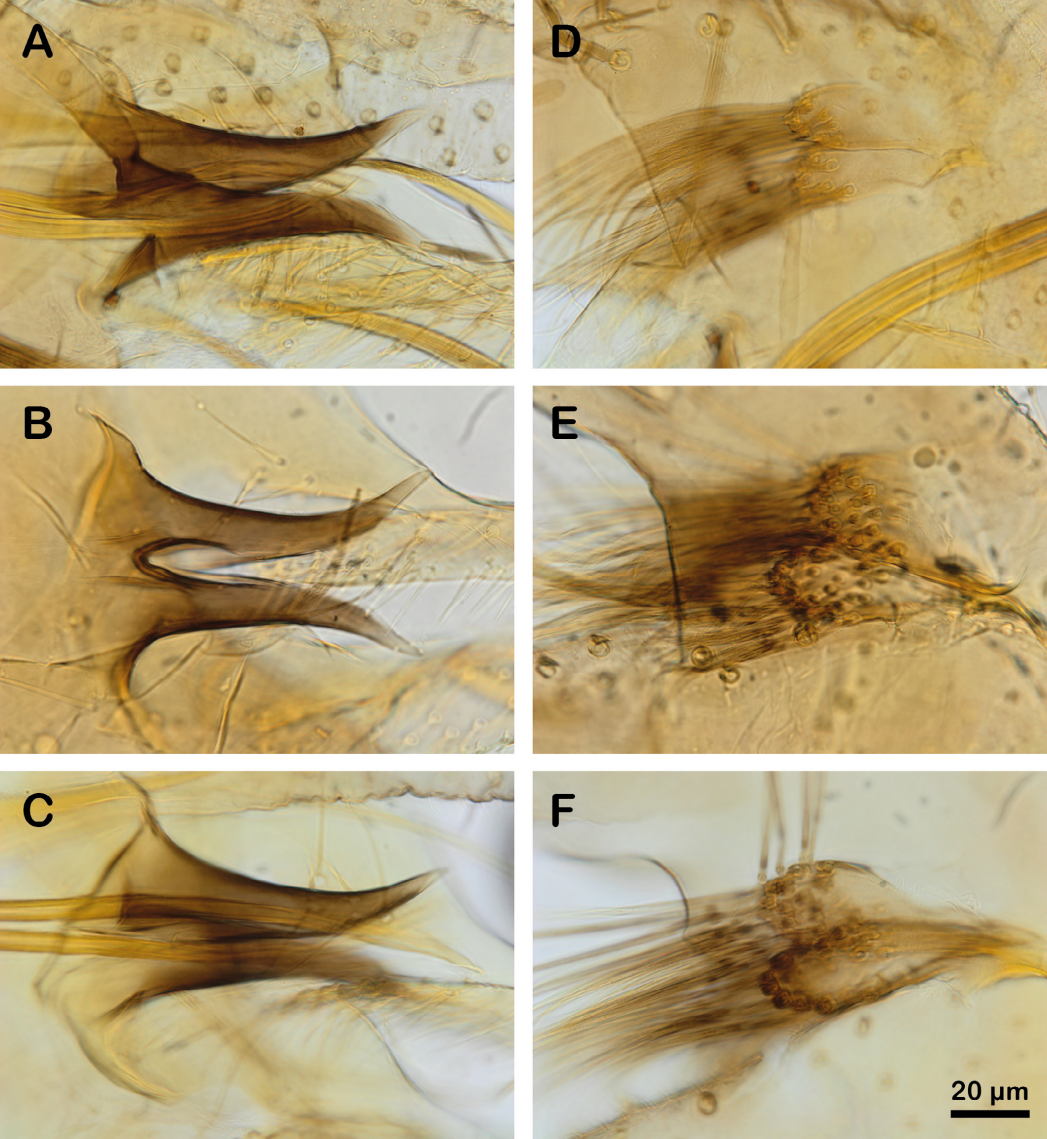
Differentiation criteria of males (A to F) 100X. A and D: aedeagus and basal lobe of coxite of *Ph. chabaudi* (SMO562); B and E: aedeagus and basal lobe of coxite of *Ph. riouxi* from Algeria (RX2); C and F: aedeagus and basal lobe of coxite of *Ph. riouxi* from Tunisia (TAT63). All photographs are set on the same scale.

*Phlebotomus chabaudi* was described for the first time in Tunisia [9] as *Paraphlebotomus* with a sharply pointed aedeagus (Figure 7, A and D). The same year, this species was also recorded in Algeria [33] and was described with a larger and more tufted basal lobe (Figure 7, E and F). The authors linked this observation to variability due to the geographically segregated populations. These two morphs have been found in sympatry without intermediate specimens [10], justifying the description of a new species: *Ph. riouxi* (Figure 7, B, C, E, F).

**Figure 8:**
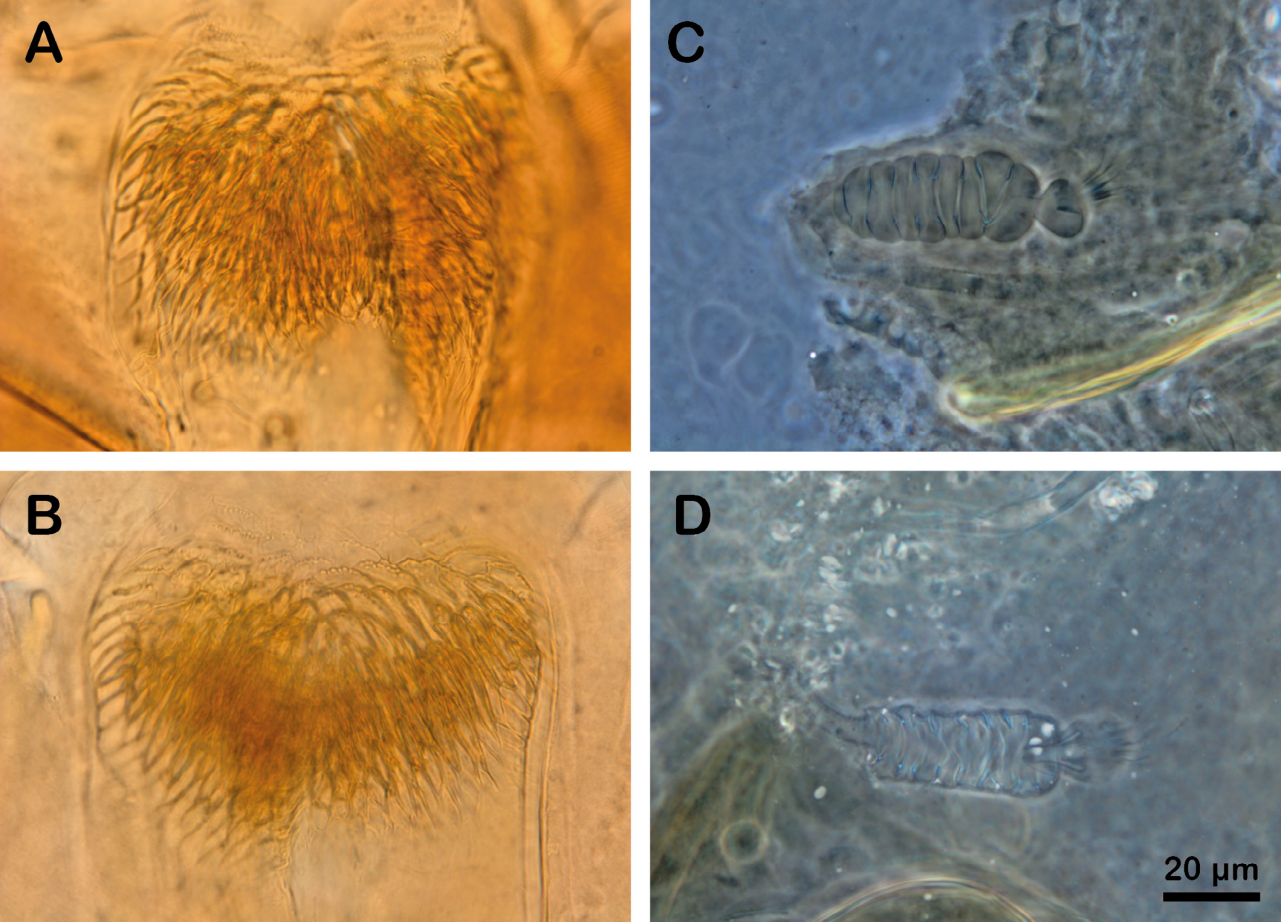
Differentiation criteria of females (A to D), 100X. A and C: pharynx and spermathecae of *Ph. chabaudi* (CBZAT583); B and D: pharynx and spermathecae of *Ph. riouxi* (TAT186 and TAT24). All photographs are set on the same scale.

The morphological identification of the female remains very difficult. It is clearly not possible to differentiate the spermathecae of the two species (Figure 8, C and D). Although Depaquit *et al.* [10] suggested examining the appearance of the armature in the genital atrium, this criterion remains uncertain. Regarding the pharynx, Bounamous *et al.* [6] noted the presence of anterolateral teeth in *Ph. chabaudi*, a character not found in *Ph. riouxi* (Figure 8, A and B). They suggested the use of this character to identify these two species, pending a larger sampling. Nevertheless, it seems that the individual variability of the pharyngeal armature of *Ph. chabaudi* makes this distinction hazardous for a non-trained entomologist.

Consequently, we selected a majority of males in the present study in order to reduce the risk of misidentification. Only a few females for which the morphological identifications were congruent with molecular analyses were included (those previously processed by Bounamous *et al.* and Boudabous *et al.* [5,6]) (Table 1). In the study by Tabbabi *et al.* [41], out of 21 specimens, two-thirds (n = 14) were morphologically identified as *Ph. riouxi* out of which 6 were males and 8 were females. Interestingly, despite the difficulty in identifying the females, all ambiguous specimens were males. Unfortunately, we could not include the specimens processed by Tabbabi in the present study and we did not find any intermediate specimens in our collection. Consequently, we performed a detailed phylogenetic analysis of *Ph. chabaudi* and *Ph. riouxi* specimens selected from our collection, and we added the sequences published by Tabbabi *et al.* [41]. Our molecular study included three independent markers. The long fragment of Cytb-CB codes for a partial protein sequence of this gene, whereas the shorter fragment (Cytb-CB3) is more frequently used for taxonomic studies [17]. Tabbabi *et al.* [40,41] analyzed the Cytb-CB fragment that they considered more informative than the Cytb-CB3 fragment. The interspecific (between closely related and/or vicariant species) and intraspecific divergence values observed for the mitochondrial cytochrome b (Cytb-CB and Cytb-CB3) in *Phlebotomus* spp. are 2.7-11% and 0.1–2.5%, respectively [12,17,27]. The 12.2% interspecific value between *Ph. chabaudi* and *Ph. riouxi* supports the genetic differentiation of these two taxa. Regarding the intraspecific values, the value for *Ph. chabaudi* (0.6%) was in the commonly accepted range. For *Ph. riouxi*, the value (4.38%) is higher than the accepted range but close to the value observed in S*ergentomyia clydei* Sinton 1928, displaying a value of 5.5% due to a divergent population from the Seychelles [32].

The nuclear EF-1α is known to be a good phylogenetic marker in Metazoa [37] and was previously used in several molecular studies in Phlebotomine sandflies [1,16,18,29,43]. Its utility in other groups has also been demonstrated in heliothine moths [8] and in Triatominae [14]. We selected this marker to compare our data with those of Tabbabi *et al.* [41]. Several studies successfully compared haplotypes of EF-1α [41,44], although EF-1α also showed considerable diversity of haplotypes for a same specimen, thus complicating the analyses [29,43]. In the present study, we also noted significant haplotype diversity. Ribosomal marker D1-D2 does not have this disadvantage. Indeed, this marker is present in many homogeneous copies in the genome, thus providing a good signal that is easy to use as a genetic marker [38]. However the nuclear ribosomal DNA may provide only a short-term marker for introgression, because of the homogenization of the multi-copy genes at this locus [15]. This marker is independent of the two previous ones and is more conserved [13]. We used this marker in previous studies and showed its usefulness for phylogenetic analysis [11,31,32].

Several ambiguous bases were observed in EF1-α sequences. Nevertheless, these ambiguous positions do not correspond to intermediate profiles between the two species. All our *Ph. riouxi* specimens from Algeria and Tunisia revealed the genotype R01 (homozygotes) corresponding to the EF_chab01 haplotype defined by Tabbabi *et al.* [41] (Table 3), in agreement with the results of Boubidi *et al.* [4]. In contrast, we obtained many new sequences of EF-1α in *Ph. chabaudi* called C01 to C16 (Table 3), providing five synapomorphic nucleotide substitutions that distinguished the two species.

Cytb and EF-1α have already been combined to demonstrate mitochondrial introgression in New World Phlebotomine sandflies [43]. Our study did not find any introgression between the two species under examination, as confirmed by the ribosomal D1-D2 analyses.

The independent phylogenetic analyses of the three genes (Figures 3, 4 and 5) underlined the subdivision of *Ph. chabaudi* and *Ph. riouxi* specimens into two independent clades. Nevertheless, our data support low genetic divergence between the two species, suggesting recent differentiation between these two taxa. This low divergence is confirmed by the low bootstrap values observed in Cytb phylogeny for the *Ph. riouxi* branch (Figure 3), in EF-1α phylogeny for the *Ph. chabaudi* branch (Figure 4), and in D1D2 phylogeny for the *Ph. chabaudi* branch (Figure 5). The phylogeny of the concatenated genes revealed bootstrap values above 70% for the two branches, suggesting that added data can only increase the differentiation between the two species.

When we consider the two phylogenies including the sequences published by Tabbabi *et al.* [41], i.e. Cytb and EF-1α phylogenies (Figures 3 and 4), all Tabbabi's sequences are included in the *Ph. riouxi* branch, without clear distinction between our specimens and Tabbabi's specimens. From these data, it is difficult to explain the disagreement between morphological characters and molecular data observed by Tabbabi *et al.* It would thus appear essential to further investigate these samples on genetic and morphological grounds to make a comparison with *Ph. chabaudi* and *Ph. riouxi* specimens from Algeria and North Tunisia.

It is worth noting that the specimens in the *Ph. riouxi* branch were only collected in Southern Algeria and Southern Tunisia, and that the specimens in the *Ph. chabaudi* branch were only collected in Northern Algeria and Northern Tunisia. This suggests a related evolution of these two taxa between the South of these two countries for *Ph. riouxi* and between the North of these two countries for *Ph. chabaudi*. The molecular clock of Cytb has been calculated for *Ph. papatasi* (Scopoli, 1786) and *Ph. duboscqi* Neveu-Lemaire 1906, two vicariant species separated by the Sahara. Its estimated calibration ranged from 1 to 2.5% variability per million years [17] or from 1.34 to 2.64% per million years [18]. *Ph. chabaudi* and *Ph. riouxi* exhibit a Cytb mean interspecific genetic divergence of 12.2%. If we apply this calibration to the latter species, we hypothesized their speciation started between 12.2 and 4.62 Mya. This period corresponds to the aridification of the Sahara desert (10 to 6 Mya). The vicariance of *Ph. chabaudi* and *Ph. riouxi* could result from the same event as the vicariance of *Ph. papatasi* and *Ph. duboscqi*. The presence of intermediate specimens as described by Tabbabi *et al.* [41], as well as specimens with morphological criteria corresponding to *Ph. chabaudi* in the South of Tunisia suggests the sympatry of the two species in Ghomrassen, which could be explained by a mixing of the two species. Only the investigation of sympatric populations will answer the unresolved question of whether or not the two lineages usually behave as true biological species when they meet. Further morphological and molecular studies on a larger sample of *Ph. riouxi* (e.g. from Ghomrassen) and on more genes remain necessary to help in determining the evolutionary history of these two species.

Finally, these results still support the existence of two species, and their typological validity, thus refuting *Ph. riouxi* as a junior synonym. The close genetic relationships and the intermediate specimens detected by Tabbabi *et al.* [41], however, suggest a recent speciation phenomenon followed by several migration events. Further genetic and morphological studies of specimens from Algeria, Tunisia and Morocco will help to better understand the evolution of these two species in North Africa.
